# Simulating the carbon balance of a temperate larch forest under various meteorological conditions

**DOI:** 10.1186/1750-0680-2-6

**Published:** 2007-05-30

**Authors:** Motomu Toda, Masayuki Yokozawa, Akihiro Sumida, Tsutomu Watanabe, Toshihiko Hara

**Affiliations:** 1 Biosphere Dynamics Research Group, Institute of Low Temperature Science, Hokkaido University, Sapporo 060-0819, Japan; 2 Department of Global Resources, National Institute for Agro-Environmental Sciences, Ibaraki 305-8604, Japan; 3 Cryosphere Environment Research Group, Institute of Low Temperature Science, Hokkaido University, Saspporo, 060-0819, Japan

## Abstract

**Background:**

Changes in the timing of phenological events may cause the annual carbon budget of deciduous forests to change. Therefore, one should take such events into account when evaluating the effects of global warming on deciduous forests. In this article, we report on the results of numerical experiments done with a model that includes a phenological module simulating the timing of bud burst and other phenological events and estimating maximum leaf area index.

**Results:**

This study suggests that the negative effects of warming on tree productivity (net primary production) outweigh the positive effects of a prolonged growing season. An increase in air temperature by 3°C (5°C) reduces cumulative net primary production by 21.3% (34.2%). Similarly, cumulative net ecosystem production (the difference between cumulative net primary production and heterotrophic respiration) decreases by 43.5% (64.5%) when temperatures are increased by 3°C (5°C). However, the positive effects of CO_2 _enrichment (2 × CO_2_) outweigh the negative effects of warming (<5°C).

**Conclusion:**

Although the model was calibrated and validated for a specific forest ecosystem, the implications of the study may be extrapolated to deciduous forests in cool-temperate zones. These forests share common features, and it can be conjectured that carbon stocks would increase in such forests in the face of doubled CO_2 _and increased temperatures as long as the increase in temperature does not exceed 5°C.

## Background

Nowadays, there is much apprehension about the great effects of global warming on the forest ecosystems, especially at mid to higher latitudes [1]. The abrupt climate change with the effect of global warming may change distributional pattern of plant species through competition among plants, and the change may then affect the global carbon cycle [2] because the vegetation ecosystems, especially forest ecosystems play a significant role in carbon exchanges between an atmosphere and biosphere [e.g.,[3-6]].

Global warming may also affect phenology of trees, especially deciduous trees. Then, the time changes in phenological events may bring about changes in the annual carbon budget in forest dynamics of the deciduous trees [7-9]. Phenology reflects the eco-physiological responses of trees that are changed by daily forcing factors such as air temperature, solar radiation and drought conditions in the atmosphere and soil. Major phenological events of deciduous woody species are the timing of bud burst (leaf unfolding), flowering and leaf-fall through leaf senescence with the course of seasons, i.e., the onset and end, respectively, of the photosynthetic active period [10-13]. A change in phenological events might be one of the most important factors affecting the future carbon sequestration potential of forests ([14,15]). In terrestrial land surfaces, percentages of deciduousness show a unimodal distribution pattern with a peak at mid-latitude, while a bimodal distributional pattern with two peaks, one at lower and the other at higher latitudes, is observed for the percentages of evergreeness [16]. However, deciduous trees are widely distributed over the temperate to boreal climate zones, adapting to the local environmental conditions (e.g., *Larix dahurica *forest stand in eastern part of Siberia). Thus, to elucidate the effects of global warming on the deciduous forests in these climate zones and the role of deciduous forests in the global carbon cycle, it is required to develop a phenological model for deciduous trees [17].

Meanwhile, the annual leaf area is also one of the most significant determinants to carbon budget. Thus we have an interest in what determines the leaf amount each year, especially maximum leaf area. The determination of maximum leaf area is closely related to phenological processes, based on the carbon reserves, and should be, therefore, considered in conjunction with the phenological events. However, an attempt has rarely been made to model the annual production of leaves (i.e. leaf area index, LAI) in deciduous trees (but see [18,19]). In most of previous atmosphere-biosphere interaction models, LAI has been given as one of assigned constants or external forcing data in relation to plant functional types (PFT) (e.g. [20-22]). For instance, Foley *et al*. [23] obtained LAI of each PFT by simply dividing leaf carbon by specific leaf area, and Sitch *et al*. [2] represented LAI by dividing leaf mass by crown area which was obtained from the allometric relationship between crown area and stem diameter. Sato, Ito and Kohyama [24] determined the maximum leaf biomass for each individual with three constraints such as crown surface area, cross-sectional area of sapwood and available resources. In addition, Ito and Oikawa [25] employed time-varying LAI so as to produce maximum daily net carbon uptake from the daily gross primary production at the entire forest stand level, which is the optimal LAI concept proposed by [26]. However, such treatments of LAI do not consider trees' physiological responses to varying environmental conditions. Aber *et al*. [18] developed a model which estimates LAI in a year using carbon reserves accumulated in buds and woody organs during several years before the corresponding year, and incorporated the framework into a forest carbon and water balance model (PnET). It might be important to consider seasonal carbon reserve mobilization for determining LAI of deciduous trees from an ecological point of view because the dynamics of carbohydrate in tree species are affected by annual variations in climate [27].

We developed a deciduous canopy development model to represent seasonal and annual dynamics of deciduous trees. The canopy development model includes a phenological model to determine the timing of bud burst and the end of the leaf appearance and a model for estimating maximum LAI. In this paper, we examined the population dynamics and carbon balance of deciduous trees by coupling the canopy development model with an integrated climate and forest dynamics model, the Multilayered Integrated Numerical Model of Surface Physics-Growing Plants Interaction (MINoSGI) [28]. MINoSGI is one of integrated atmosphere-forest dynamics interaction models. The model describes population dynamics of trees or growth (carbon sink) and death (carbon source) of individual trees which are relevant to carbon dynamics in a forest by using a partial differential equation for the size distribution density [28-30]. Thus, MINoSGI is an alternative approach to individually based vegetation dynamics models which resolve spatial locations of individual plants to mimic horizontal interference between individuals [e.g., [31,24]]. Here, the original algorithm of MINoSGI, which had been applicable only to evergreen trees, was further extended to be applicable to deciduous trees by incorporating the above-mentioned features with respect to the timing of bud burst and the seasonal carbon storage dynamics of deciduous trees. The model was applied to a deciduous coniferous *Larix kaempferi *forest in a climatologically temperate area, and its performance tested by comparison with inventory data.

Further, sensitivity analysis was conducted to assess an individual or combined effect of increased CO_2 _concentration and air temperature on forest dynamics and production of a forest over a hundred years. The objective is to examine the responses of size-structure dynamics of a plant community to changes in exogenous factors such as CO_2 _concentration, air temperature. We emphasize that it is not intended to project impacts of future climate change on plant communities and global carbon cycle. Therefore, the sensitivity analysis is the first step to evaluate the impact of predicted changes in climate conditions such as air temperature and atmospheric CO_2 _concentration with the effect of global warming on forest production along with variations in size structure of the deciduous coniferous forest on a long time scale.

## Results

### Simulated biomass, averaged tree height and organic matter production

**Figure 1:**
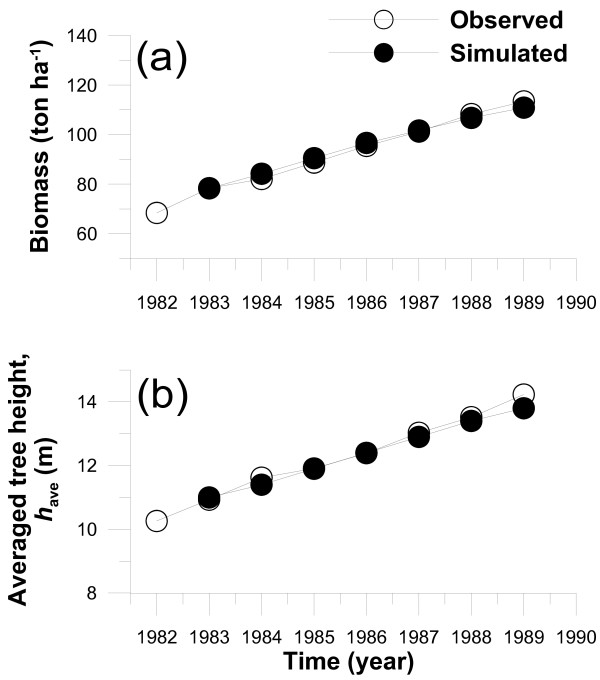
Observed and simulated annual variations in (a) forest stand biomass and (b) averaged tree height (*h*_ave_) in the *Larix kaempferi *stand.

It was examined whether the model with the adjusted parameters (see the Methods section in details) produces realistic population dynamics of the forest over seven years. The simulated annual variations in the above-ground biomass and averaged tree height were reproduced quite well by simulation (Fig. 1). The observed and simulated biomass and averaged tree height were 113, 111 ton ha^-1 ^and 14.2, 14.5 m in 1989, respectively.

**Figure 2:**
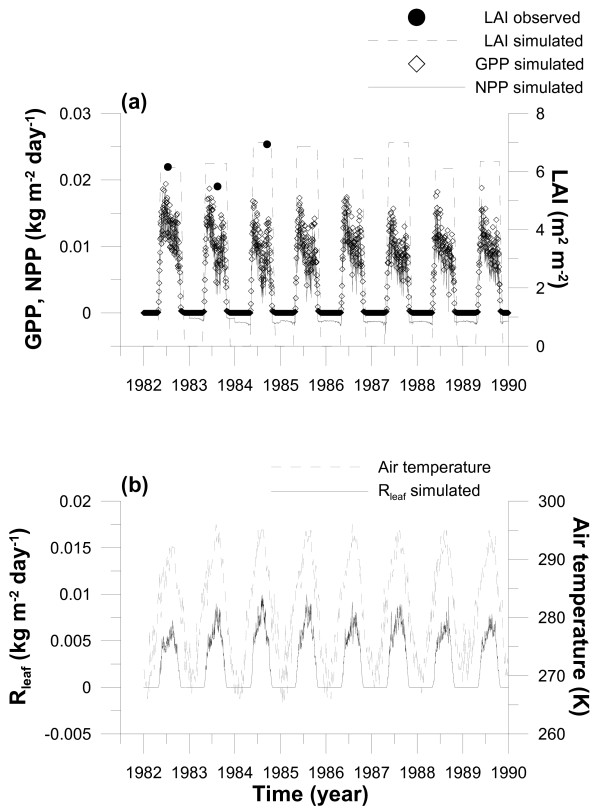
(a) Simulated seasonal and annual variations in gross primary production (GPP) and net primary production (NPP) and LAI in the *Larix kaempferi *stand. The vertical axes on the left- and right-hand sides are employed for GPP, NPP and LAI, respectively. LAIs observed in 1982, 1983 and 1984 are also plotted in this figure. (b) Simulated seasonal and annual variations in leaf respiration (R_leaf_) and air temperature. The vertical axes on the left- and right-hand sides are employed for R_leaf _and air temperature, respectively.

The simulated annual gross primary production (GPP) and net primary production (NPP) within the forest in our study over 7 years were 32.6 and 12.1 on average in the range from 31.8 to 33.9 and from 10.8 to 14.7 (ton ha^-1 ^year^-1^), respectively (Fig. 2(a)). Both GPP and NPP have a similar seasonal trend, i.e., two peaks in spring and autumn. A large decrease in GPP and NPP was shown in summer. The decrease was caused by the increase in leaf respiration (R_leaf_) which is mainly controlled by a seasonal change in air temperature (Fig. 2(b)). R_leaf _was seven times as large as woody organ's respiration rates (stem and root) and 1.8 times larger than soil respiration on average. Therefore, averaged NPP/GPP was 0.37. In addition, the NPP simulated in the present study was consistent with the results referred to in [32], who summarized NPPs estimated in many *Larix *forests in the cool-temperate zone in China, and was equivalent to those obtained in other deciduous forests (e.g., 10 ton ha^-1 ^year^-1 ^in the case of a mixed forest which consisted of sugar maple, American beech, and yellow birch in [33]).

### Sensitivity to meteorological conditions

**Figure 3:**
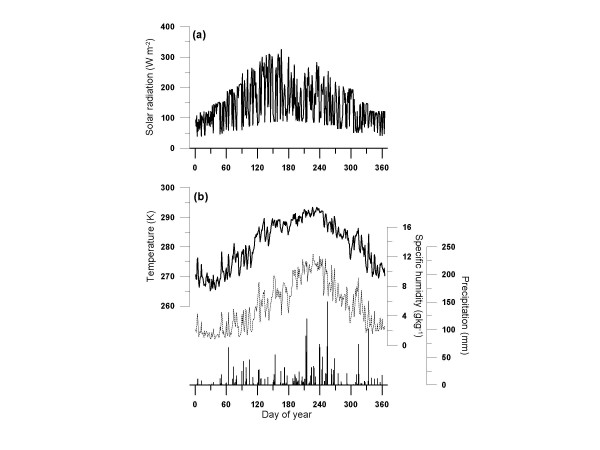
Seasonal variations in meteorological variables on a daily mean basis; (a) solar radiation (Wm^-2^), (b) air temperature (K), specific humidity (kgkg^-1^) and precipitation obtained at the AMEDAS meteorological data station of Inabu, Japan. The annual dataset with 150 repetitions was used as part of meteorological forcing data.

Changes in the future climate system might affect the dynamics of forests that are generated by competition for resources between individual trees. Then, dynamic changes of forests have a possibility to produce variations in carbon balance of the forests. Herein, with the fully validated MINoSGI model, we aimed at sensitivity analysis to assess how changes in air temperature and atmospheric CO_2 _concentration affect production and size structure of a *L. kaempferi *forest with forest development. In the simulation, we assumed a monospecific *L. kaempferi *forest which was planted at a density of 5000 trees per hectre. The initial sapling size in the stand was assumed to be uniform and was set at the smallest height class at the beginning of the simulation. In addition, we used an annual meteorological dataset with 150 repetitions, which was generated using the data in 1986 from the Inabu experimental site ([34]; Fig. 3) in order to exclude the effect of year-to-year variations in meteorological forcing data on production of the *Larix *forest. In addition, we assumed that the values of initial carbon stock in each component in the soil carbon dynamics model were identical to those used in the model validation for the *Larix *forest site in Inabu experimental field [See the Methods section]. Eight simulations were made under the following conditions: (1) the present climate conditions (control; see Fig. 3), (2) 3°C increase in air temperature (+3°C), (3) 5°C increase in air temperature (+5°C), (4) 10°C increase in air temperature (+10°C), (5) doubled CO_2 _concentration (2 × CO_2_), (6) combined effect of (2) and (5), (7) combined effect of (3) and (5), (8) combined effect of (4) and (5). The annual averaged values of daily mean solar radiation, air temperature, specific humidity and annual amount of precipitation were 145 Wm^-2^, 7.7°C, 5.4 kgkg^-1 ^and 2256 mm, respectively (Fig. 3). The precipitation accumulated during summer (from July to September) was 1196 mm, representing more than a half of the annual amount (Fig. 3).

**Figure 4:**
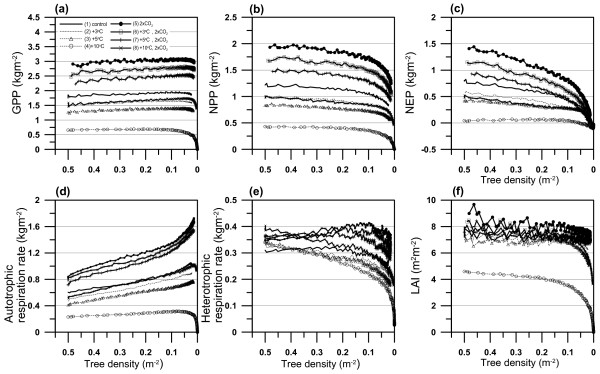
Simulated annual variations in forest production; (a) gross primary production (GPP), (b) net primary production (NPP), (c) net ecosystem production (NEP), (d) autotrophic respiration rate (R_a_), (e) heterotrophic respiration rate (R_h_) and (f) leaf area index (LAI) with the decrease in tree density (forest stand development). Eight simulations were conducted under the following conditions: (1) the present climate conditions (control; see Fig. 7), (2) 3°C increase in air temperature (+3°C), (3) 5°C increase in air temperature (+5°C), (4) 10°C increase in air temperature (+10°C), (5) doubled CO_2 _concentration (2 × CO_2_), (6) combined effect of (2) and (5), (7) combined effect of (3) and (5), (8) combined effect of (4) and (5).

We can see similar annual trends of slightly increasing or almost constant GPP and decreasing NPP and NEP (net ecosystem production) with the decrease in tree density, namely, with forest development, except for the one with the individual effect of increased air temperature by 10°C (Fig. 4(a)–(c)). In particular, a large decrease in annual NPP with forest stand development occurred in the seven simulations due to a large increase in autotrophic respiration rate (*R*_a_) relative to GPP with tree growth in 2 × CO_2 _condition. *R*_a _was increased with forest development in the seven simulations due to the increment of sapwood and root biomass with tree growth from seedling (Fig. 4(d)). In contrast, heterotrophic respiration rate (*R*_h_) with forest development got greater as air temperature increased (Fig. 4(e)). *R*_h _is more strongly influenced by the litter decomposition pool with greater decomposition rate than by the remainder soil organic matter (SOM) pools with smaller decomposition rate. The amount of litter supply from above-ground into the litter decomposition pool appears to be closely related to the size of trees. LAI gradually decreased with forest development in all the eight simulations. LAI at a specific tree density (i.e. forest development) decreased in the order of 2 × CO_2_, +3°C and 2 × CO_2_, +5°C and 2 × CO_2_, control, +10°C and 2 × CO_2_, +3°C and +5°C, the same as in GPP, NPP and NEP. The great decrease in LAI under lower tree densities was caused by the individual effect of increased temperature which yielded a number of dead trees (Fig. 4(f)).

GPP, NPP and NEP in 2 × CO_2 _were greater than those in other conditions. 2 × CO_2 _enhanced the growth of trees with forest development and produced the greatest averaged tree height, stem diameter and LAI in all the conditions (Fig. 4(f)) and then brought about increases in the cumulative NPP and NEP by 55.9% and 53.2%, respectively, over the 150-year period, compared with those under the control conditions. Furthermore, the decreases in annual NPP and NEP with forest development when tree density was less than 0.1 were the largest in 2 × CO_2_, and annual NPP and NEP after 150 years from the start of the simulation were 1.24 and 0.15 kgm^-2^. These values represented decreases by 30% and 74% as compared with those obtained at the final time step when tree density was assumed to be equal to the initial one, which is identical to the output from static land surface models that do not consider forest dynamics.

**Figure 5:**
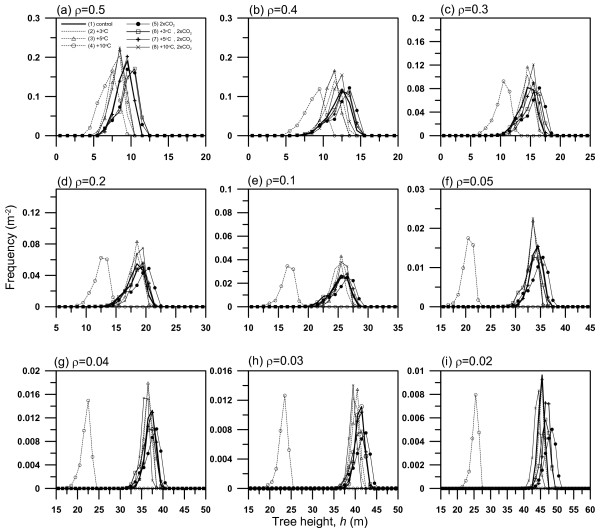
Size distributions obtained from the eight simulations in Fig. 4 at 9 specific tree densities from the initial tree density ρ = 0.5 to ρ = 0.02.

In 2 × CO_2_, the growth of all trees was enhanced and a large difference in the growth rate between large- and small-sized trees was also induced (Fig. 5). During the period with tree densities greater than 0.1, intensive tree competition reduced tree density with time, and a number of smaller-sized trees in the stand died gradually due to the light shade effect of larger-sized ones. However, at tree densities less than 0.1, the relative growth rate of larger-sized trees in the forest was greatly reduced by the decreased carbon assimilation essential to their growth and the increased *R*_a _(Fig. 4(d)). Further, *R*_h _was decreased during the corresponding period due to a slight supply of leaf-fall from living larger-sized trees. Hence, we see deaths of larger-sized trees and the great reduction of annual NPP and NEP. This was because of the increase in vertically overlapped leaves between smaller- and larger-sized trees as a consequence of large height-growth of smaller-sized trees. During the later simulation period, the height of larger-sized trees in the stand exceeded 50 m. Accordingly, due to the deaths of larger-sized trees the reduction of annual NPP and NEP in 2 × CO_2 _was the greatest in all the conditions.

On the other hand, an individual effect of increased air temperature (+3°C, +5°C, +10°C) brought about decreased NPP and NEP compared with the control conditions. The increase in air temperature brought about earlier start of bud burst and thus the increase in the period of photosynthetically active leaves. In +3°C and 5°C bud burst was earlier by 15.6 days (97.7 days from Jan. 1) and 26.8 days (86.4 days from Jan. 1) than that in the control conditions (113 days from Jan. 1) on average throughout the simulation period. Nevertheless, increased temperature did not enhance the growth of trees, especially the growth rate of trees in +10°C was much smaller than that in other conditions (Fig. 5) and all the trees died at year 123 from the start of the simulation period. This suggests that the negative effect of an increase in respiration by increased temperature on NPP and NEP dominates over the positive effect of prolonged growing season on NPP and NEP. Hence, the magnitude and annual variations in NPP and NEP were reduced by increased temperature (Fig. 4(b), (c)). On the other hand, *R*_h _decreased as air temperature increased, and *R*_h _did not increase with forest stand development because trees with smaller growth rates did not much contribute to the litter decomposition pool. The increase in air temperature by 3°C or 5°C brought about the reductions of 21.3% and 34.2%, 43.5% and 64.5% in cumulative NPP and NEP, respectively, over the 150-year simulation period, compared with those under the control conditions. Furthermore, cumulative NPP decreased by 440% in +10°C compared with the control conditions, and cumulative NEP was negative, indicating that the forest worked as carbon source.

The combined effects of increased temperatures and doubled CO_2 _concentration produced intermediate production between those in each individual effect, and NPP got smaller as air temperature increased in 2 × CO_2 _conditions. In 2 × CO_2 _and +3°C, cumulative NPP and NEP increased by 35.2% and 11.9% over the 150-year simulation period, compared with those under the control conditions. These increases in NPP and NEP indicate that the positive fertilization effect of 2 × CO_2 _on NPP and NEP dominated over the negative effect (due to increased respiration) of increased air temperature by <5°C. In contrast, cumulative NPP increased by 18%, but cumulative NEP decreased 14.4% in 2 × CO_2 _and +5°C compared with the control conditions. Furthermore, cumulative NPP and NEP decreased by 33.4% and 109.5% in 2 × CO_2 _and +10°C compared with the control conditions. This indicates that the negative effect of increased air temperature by ≥ 5°C on NEP dominated over the positive effect of 2 × CO_2_.

## Discussion

A lack of the understanding of plant acclimation to future climate change makes it difficult to elucidate the role of the forest in global carbon cycle on a long time scale. Furthermore, the lack produces uncertainties of the sensitivity tests of plant responses to doubled CO_2 _concentration and increased air temperature conditions. Recently, Körner [35] has comprehensively summarized the results of plant CO_2 _responses obtained from CO_2_-enrichment experiments and represented that elevated CO_2 _concentration is beneficial for plant growth irrespective of species and experimental designs. Some experimental reports showed that elevated CO_2 _concentration stimulated strongly only initial growth of trees only, whereas such plant CO_2 _responses declined or disappeared after several years [36,37]. The difference in plant CO_2 _responses with age has probably arisen from acclimation of trees to the elevated CO_2 _concentration. Our sensitivity tests did not consider the plant acclimation. Therefore, the predicted results may be different from the above-mentioned experimental ones concerning the impacts of elevated CO_2 _concentration and increased air temperature on the production of a deciduous forest.

## Conclusion

It is well-known that rising CO_2 _concentration has been caused by anthropogenic emissions. Simultaneously, the climate change due to the rising CO_2 _concentration should accelerate carbon emission from natural forest ecosystems into the atmosphere [38] which means positive feedback of the release of carbon into the atmosphere. It was suggested that global carbon cycle is strongly controlled by soil decomposition processes of soil organic carbon and it is important to fully consider soils in assessing the full impact of climate change through a terrestrial carbon cycle modeling approach [39,40]. Accordingly, forest modeling is expected to be used as a tool for policy decision-makers in order to predict the effect of climate change on carbon stocks in the forest ecosystems and to plan the most sustainable management under climate change [41].

For this purpose, a canopy development model for phenological processes of the timing of bud burst and for the estimation of annual variations in leaf amount (LAI) by considering seasonal carbon reserve was designed. This model was coupled with the integrated climate and forest dynamics model (MINoSGI), and its application to a deciduous *L. kaempferi *forest was examined for stand-level variables such as biomass, averaged tree height and NPP. Although the present model deals with averaged size-dependence of individual growth in a limited one-dimensional framework, our model simulation yielded realistic forest dynamics over seven years as the result of careful parameter adjustment based on the inventory data.

The agreement between observed and simulated values of biomass and averaged tree height enhanced the reliability of MINoSGI, whereas a discrepancy between the observed and simulated timing of bud burst in 1983 occurred. Several species-specific parameters relevant to the timing of bud burst in the phenological model should be determined from experimental and statistical approaches, which will be conducted in the following study. Furthermore, in MINoSGI, daily photosynthetic rate was determined as a result of energy, water and carbon exchanges between the atmosphere and forest as affected by variations in meteorological and hydrological conditions such as light, temperature and soil water from hour to hour. The phenological framework of the present study that an accumulation of the assimilated carbon into the carbon pool at the end of growing season is used to build leaves only for the next year is not necessarily true of all species. Hence, further implementation of the determination of maximum LAI relevant to the seasonal cycle of carbohydrate mobilization in the tree body might be required in the present phenological scheme, by considering species-specific features.

Numerical experiments confirmed the importance of the present canopy development model, i.e. determining the annual variation in LAI of a deciduous forest based on the carbohydrate reserve and mobilization, and has enhanced the possible utility of the forest dynamics model, MINoSGI, coupled with the canopy development model.

Furthermore, with the fully validated model, we found that doubled CO_2 _concentration increased and increased air temperature reduced the production of a deciduous forest over the 150-year simulation period. Hence, the sensitivity test suggests that the deciduous forest ecosystem in the cool-temperate climate zone has enhanced efficiency of carbon absorption over a hundred years in doubled CO_2 _at increased temperatures by <5°C compared with the present climate conditions but deteriorated one in doubled CO_2 _concentration at increased temperatures by ≥ 5°C. The former is due to the CO_2 _fertilization effect that surpasses the increased temperature's negative effect on production (i.e. respiration is increased by temperature), the latter vice versa.

As a next step, using the improved MINoSGI coupled with the canopy development model with variable LAI as affected by varying environments, we can predict variations in the annual carbon budget of the deciduous forests in the cool-temperate and boreal climate zones, where there is great apprehension about the effects of global warming on forest ecosystems, with climate change scenarios. Therefore, we can expect not only to elucidate the role of deciduous forests of these regions in the global carbon cycle but also to apply our model to assessing of the effects of forest management and climate change on the carbon stocks in managed and unmanaged (or natural) forest ecosystems.

## Methods

### 1. MINoSGI

For the purpose of describing the interactions between meteorological physics and the growth of plants with the progress of time, Watanabe *et al*. [28] developed a gap model combined with a multilayered microclimate environment and forest dynamics, MINoSGI. MINoSGI consists of two submodels, i.e., a plant size dynamics model and a micrometeorological model, and simulates dynamics such as the change of plant growth and mortality due to competition between individual trees within a given physical environment inside a forest. Such dynamics of the forest, in turn, cause changes in the physical environment. This interactive feedback process allows us to predict the impact of climatic change on forest dynamics on a long time scale. Watanabe *et al*. [28] applied MINoSGI to an evergreen coniferous Sugi stand (*Cryptomeria japonica *D. Don, the Japanese cedar, Taxodiaceae) in monoculture, and was successful in reproducing the size-structure dynamics of the forest over six years in comparison with ecological inventory data obtained from 1983 to 1988 [42]. For the purpose of prognosticating physical variables such as soil temperature and moisture, snow mass, snow surface temperature, snow albedo and runoff in the soil ground process, MINoSGI includes the ground process scheme similar to MATSIRO (Minimal Advanced Treatments of Surface Interaction and RunOff), which is a land surface model developed to be coupled with an atmospheric general circulation model [43]. In the ground process scheme, five vertical multi-layered distributions of soil temperature and moisture are prognosticated in the soil for a whole forest stand. Snow surface temperature and albedo on the ground are also predicted by this model. These are calculated from the input variables such as nonconvective rainfalls and snowfalls using the fluxes in MINoSGI at hourly time steps. With prognosticated soil moisture, soil water stress was calculated in accordance with a stress function due to soil water deficit defined by [44]. Thus, the inclusion of soil water stress in the ground process scheme into MINoSGI affects daily leaf photosynthesis, namely, the height- or diameter-growth of trees under a specific soil moisture condition. The treatment can be acceptable from the observational result that the increment of diameter growth of trees is constrained by soil moisture deficit [45].

The fundamental treatment to predict forest dynamics in MINoSGI is described as follows. In MINoSGI, the prognostic equations predicting the dynamics of a distribution density *f*(*t*,*h*) of individuals and the total plant mass *f(t,h)w(t,h) *of plant height class *h *per unit ground area at time *t *are given by

∂f(t,h)∂t=12∂2∂h2{D(t,h)f(t,h)}−∂∂h{G(t,h)f(t,h)}−M(t,h)f(t,h),
 MathType@MTEF@5@5@+=feaafiart1ev1aaatCvAUfKttLearuWrP9MDH5MBPbIqV92AaeXatLxBI9gBaebbnrfifHhDYfgasaacH8akY=wiFfYdH8Gipec8Eeeu0xXdbba9frFj0=OqFfea0dXdd9vqai=hGuQ8kuc9pgc9s8qqaq=dirpe0xb9q8qiLsFr0=vr0=vr0dc8meaabaqaciaacaGaaeqabaqabeGadaaakeaadaWcaaqaaiabgkGi2kabdAgaMnaabmaabaGaemiDaqNaeiilaWIaemiAaGgacaGLOaGaayzkaaaabaGaeyOaIyRaemiDaqhaaiabg2da9maalaaabaGaeGymaedabaGaeGOmaidaamaalaaabaGaeyOaIy7aaWbaaSqabeaacqaIYaGmaaaakeaacqGHciITcqWGObaAdaahaaWcbeqaaiabikdaYaaaaaGcdaGadaqaaiabdseaenaabmaabaGaemiDaqNaeiilaWIaemiAaGgacaGLOaGaayzkaaGaemOzay2aaeWaaeaacqWG0baDcqGGSaalcqWGObaAaiaawIcacaGLPaaaaiaawUhacaGL9baacqGHsisldaWcaaqaaiabgkGi2cqaaiabgkGi2kabdIgaObaadaGadaqaaiabdEeahnaabmaabaGaemiDaqNaeiilaWIaemiAaGgacaGLOaGaayzkaaGaemOzay2aaeWaaeaacqWG0baDcqGGSaalcqWGObaAaiaawIcacaGLPaaaaiaawUhacaGL9baacqGHsislcqWGnbqtdaqadaqaaiabdsha0jabcYcaSiabdIgaObGaayjkaiaawMcaaiabdAgaMnaabmaabaGaemiDaqNaeiilaWIaemiAaGgacaGLOaGaayzkaaGaeiilaWcaaa@72CD@

and

∂f(t,h)w(t,h)∂t=Pn(t,h)f(t,h)+12∂2∂h2{D(t,h)f(t,h)w(t,h)}−∂∂h{G(t,h)f(t,h)w(t,h)}−M(t,h)f(t,h)w(t,h)−f(t,h)[Lf(t,h)+Lw(t,h)+Lr(t,h)],
 MathType@MTEF@5@5@+=feaafiart1ev1aaatCvAUfKttLearuWrP9MDH5MBPbIqV92AaeXatLxBI9gBaebbnrfifHhDYfgasaacH8akY=wiFfYdH8Gipec8Eeeu0xXdbba9frFj0=OqFfea0dXdd9vqai=hGuQ8kuc9pgc9s8qqaq=dirpe0xb9q8qiLsFr0=vr0=vr0dc8meaabaqaciaacaGaaeqabaqabeGadaaakeaafaqadeWabaaabaWaaSaaaeaacqGHciITcqWGMbGzdaqadaqaaiabdsha0jabcYcaSiabdIgaObGaayjkaiaawMcaaiabdEha3naabmaabaGaemiDaqNaeiilaWIaemiAaGgacaGLOaGaayzkaaaabaGaeyOaIyRaemiDaqhaaiabg2da9iabdcfaqnaaBaaaleaacqWGUbGBaeqaaOWaaeWaaeaacqWG0baDcqGGSaalcqWGObaAaiaawIcacaGLPaaacqWGMbGzdaqadaqaaiabdsha0jabcYcaSiabdIgaObGaayjkaiaawMcaaaqaaiabgUcaRmaalaaabaGaeGymaedabaGaeGOmaidaamaalaaabaGaeyOaIy7aaWbaaSqabeaacqaIYaGmaaaakeaacqGHciITcqWGObaAdaahaaWcbeqaaiabikdaYaaaaaGcdaGadaqaaiabdseaenaabmaabaGaemiDaqNaeiilaWIaemiAaGgacaGLOaGaayzkaaGaemOzay2aaeWaaeaacqWG0baDcqGGSaalcqWGObaAaiaawIcacaGLPaaacqWG3bWDdaqadaqaaiabdsha0jabcYcaSiabdIgaObGaayjkaiaawMcaaaGaay5Eaiaaw2haaiabgkHiTmaalaaabaGaeyOaIylabaGaeyOaIyRaemiAaGgaamaacmaabaGaem4raC0aaeWaaeaacqWG0baDcqGGSaalcqWGObaAaiaawIcacaGLPaaacqWGMbGzdaqadaqaaiabdsha0jabcYcaSiabdIgaObGaayjkaiaawMcaaiabdEha3naabmaabaGaemiDaqNaeiilaWIaemiAaGgacaGLOaGaayzkaaaacaGL7bGaayzFaaaabaGaeyOeI0Iaemyta00aaeWaaeaacqWG0baDcqGGSaalcqWGObaAaiaawIcacaGLPaaacqWGMbGzdaqadaqaaiabdsha0jabcYcaSiabdIgaObGaayjkaiaawMcaaiabdEha3naabmaabaGaemiDaqNaeiilaWIaemiAaGgacaGLOaGaayzkaaGaeyOeI0IaemOzay2aaeWaaeaacqWG0baDcqGGSaalcqWGObaAaiaawIcacaGLPaaadaWadaqaaiabdYeamnaaBaaaleaacqWGMbGzaeqaaOWaaeWaaeaacqWG0baDcqGGSaalcqWGObaAaiaawIcacaGLPaaacqGHRaWkcqWGmbatdaWgaaWcbaGaem4DaChabeaakmaabmaabaGaemiDaqNaeiilaWIaemiAaGgacaGLOaGaayzkaaGaey4kaSIaemitaW0aaSbaaSqaaiabdkhaYbqabaGcdaqadaqaaiabdsha0jabcYcaSiabdIgaObGaayjkaiaawMcaaaGaay5waiaaw2faaiabcYcaSaaaaaa@BFE9@

where *w(t,h) *is mean plant mass of each *h*, *D(t,h)*, *G(t,h) *and *M(t,h) *are variance and mean of growth rate in height, and mortality rate of individuals, respectively. *P*_*n*_*(t,h) *is the net primary production (NPP) per plant of each plant height class and *L*_*f*_*(t,h)*, *L*_*w*_*(t,h) *and *L*_*r*_*(t,h) *are the litter production rate for leaf, wood and fine roots, respectively. These equations include the original diffusion model proposed by [29], which describes fluctuations caused by various factors such as spatial heterogeneity of environmental variables, spatial variation of individuals in the neighborhood competitive effects [29,46]. However, in the present study, the diffusion term *D(t,h) *in eqns. (1) and (2) is excluded because it is assumed that the experimental forest area is covered by a spatially homogeneous land surface.

In MINoSGI, the vertical distribution of leaf area is estimated from the pipe-model theory proposed by [47], which relates leaf area of plant height *h *and stem diameter *d *of a plant. The vertical profile of stem diameter of plant height *h*, *d(t,z,h)*, is described as follows:

d(t,z,h)=d0(t,h)[1−(zh)η],
 MathType@MTEF@5@5@+=feaafiart1ev1aaatCvAUfKttLearuWrP9MDH5MBPbIqV92AaeXatLxBI9gBaebbnrfifHhDYfgasaacH8akY=wiFfYdH8Gipec8Eeeu0xXdbba9frFj0=OqFfea0dXdd9vqai=hGuQ8kuc9pgc9s8qqaq=dirpe0xb9q8qiLsFr0=vr0=vr0dc8meaabaqaciaacaGaaeqabaqabeGadaaakeaacqWGKbazdaqadaqaaiabdsha0jabcYcaSiabdQha6jabcYcaSiabdIgaObGaayjkaiaawMcaaiabg2da9iabdsgaKnaaBaaaleaacqaIWaamaeqaaOWaaeWaaeaacqWG0baDcqGGSaalcqWGObaAaiaawIcacaGLPaaadaWadaqaaiabigdaXiabgkHiTmaabmaabaWaaSaaaeaacqWG6bGEaeaacqWGObaAaaaacaGLOaGaayzkaaWaaWbaaSqabeaacqaH3oaAaaaakiaawUfacaGLDbaacqGGSaalaaa@493C@

Where z is height from the ground, *d*_0 _*(t,h) *is stem diameter at the ground and η is a parameter. As η increases, the shape of stem changes from conic to spheroidal [48]. With the stem diameter distribution, the vertical distribution of leaf area is given by

FL(t,z,h)=∂(θd2)∂z=2θηd02[1−(zh)η]zη−1hη
 MathType@MTEF@5@5@+=feaafiart1ev1aaatCvAUfKttLearuWrP9MDH5MBPbIqV92AaeXatLxBI9gBaebbnrfifHhDYfgasaacH8akY=wiFfYdH8Gipec8Eeeu0xXdbba9frFj0=OqFfea0dXdd9vqai=hGuQ8kuc9pgc9s8qqaq=dirpe0xb9q8qiLsFr0=vr0=vr0dc8meaabaqaciaacaGaaeqabaqabeGadaaakeaacqWGgbGrdaWgaaWcbaGaemitaWeabeaakmaabmaabaGaemiDaqNaeiilaWIaemOEaONaeiilaWIaemiAaGgacaGLOaGaayzkaaGaeyypa0ZaaSaaaeaacqGHciITdaqadaqaaGGaciab=H7aXjabdsgaKnaaCaaaleqabaGaeGOmaidaaaGccaGLOaGaayzkaaaabaGaeyOaIyRaemOEaOhaaiabg2da9iabikdaYiab=H7aXjab=D7aOjabdsgaKnaaDaaaleaacqaIWaamaeaacqaIYaGmaaGcdaWadaqaaiabigdaXiabgkHiTmaabmaabaWaaSaaaeaacqWG6bGEaeaacqWGObaAaaaacaGLOaGaayzkaaWaaWbaaSqabeaacqWF3oaAaaaakiaawUfacaGLDbaadaWcaaqaaiabdQha6naaCaaaleqabaGae83TdGMaeyOeI0IaeGymaedaaaGcbaGaemiAaG2aaWbaaSqabeaacqWF3oaAaaaaaaaa@5D10@

with the allometric parameter *θ *and the vertical distribution of potential leaf area of a height class *h*, *F*_*L*_*(t,z,h)*. In a dense forest, the actual leaf amount at the bottom of the canopy layer is reduced by competition between individuals, i.e., self-pruning or the rising of crown. Watanabe *et al*. [28] calculated the actual leaf area *F*_*LA*_*(t,z,h) *by incorporating the temporal change of leaf expansion coefficient for each layer at daily time steps based on a leaf carbon budget. When a negative carbon budget per leaf of a specific layer is calculated, *F*_*LA*_*(t,z,h) *at the layer is estimated by reducing the leaf amount of the corresponding dry matter by the negative carbon amount. In the framework of this study, *F*_*L *_*(t,z,h) *is calculated by considering the difference between maximum tree height of a height class and the height of the mean crown base *z*_*cb*_, which is decided as the height after the occurrence of the crown rise due to the performance of dead leaves. Using this *F*_*L *_*(t,z,h*) as the upper limited leaf area, the actual maximum leaf area *F*_*LA*_*(t,z,h) *is determined through the comparison with leaf area, which is produced by phenological processes in which the leaf area is determined by using carbon assimilation by tree leaves stored during the latter part of the growing season until defoliation to build a leaf amount for the next year.

The model parameters, which cannot be determined because of the lack of observational approaches and/or are undocumented, are adjusted into formulated relationships using inventory data. The vertical profile of crown diameter *d*_*crown *_is calculated as

dcrown(t,z,h)=δd0(1−zh)12
 MathType@MTEF@5@5@+=feaafiart1ev1aaatCvAUfKttLearuWrP9MDH5MBPbIqV92AaeXatLxBI9gBaebbnrfifHhDYfgasaacH8akY=wiFfYdH8Gipec8Eeeu0xXdbba9frFj0=OqFfea0dXdd9vqai=hGuQ8kuc9pgc9s8qqaq=dirpe0xb9q8qiLsFr0=vr0=vr0dc8meaabaqaciaacaGaaeqabaqabeGadaaakeaacqWGKbazdaWgaaWcbaGaem4yamMaemOCaiNaem4Ba8Maem4DaCNaemOBa4gabeaakmaabmaabaGaemiDaqNaeiilaWIaemOEaONaeiilaWIaemiAaGgacaGLOaGaayzkaaGaeyypa0dcciGae8hTdqMaemizaq2aaSbaaSqaaiabicdaWaqabaGcdaqadaqaaiabigdaXiabgkHiTmaalaaabaGaemOEaOhabaGaemiAaGgaaaGaayjkaiaawMcaamaaCaaaleqabaWaaSGaaeaacqaIXaqmaeaacqaIYaGmaaaaaaaa@4A56@

with the adjusted parameter *δ*, stem diameter at the ground *d*_0 _and distance from the ground level *z*. The next adjusted parameter *f*_*d *_is used for the relationship between leaf respiration rate *R*_*d *_and maximum catalytic activity of Rubisco *V*_*m *_as follows [28]

*R*_*d *_= *f*_*d*_*V*_*m*_.

In addition, we considered the allocation relationship between plant height *h *and stem diameter *d*_0 _in each height class. The difference in allocation between individual tree height classes might produce different competition modes, resulting in different forest dynamics. Watanabe *et al*. [28] introduced the following relationship on the basis of [49]:

ΔhΔd0=μ0+μ1(1Im(t,h)−1),
 MathType@MTEF@5@5@+=feaafiart1ev1aaatCvAUfKttLearuWrP9MDH5MBPbIqV92AaeXatLxBI9gBaebbnrfifHhDYfgasaacH8akY=wiFfYdH8Gipec8Eeeu0xXdbba9frFj0=OqFfea0dXdd9vqai=hGuQ8kuc9pgc9s8qqaq=dirpe0xb9q8qiLsFr0=vr0=vr0dc8meaabaqaciaacaGaaeqabaqabeGadaaakeaadaWcaaqaaiabfs5aejabdIgaObqaaiabfs5aejabdsgaKnaaBaaaleaacqaIWaamaeqaaaaakiabg2da9GGaciab=X7aTnaaBaaaleaacqaIWaamaeqaaOGaey4kaSIae8hVd02aaSbaaSqaaiabigdaXaqabaGcdaqadaqaamaalaaabaGaeGymaedabaGaemysaK0aaSbaaSqaaiabd2gaTbqabaGcdaqadaqaaiabdsha0jabcYcaSiabdIgaObGaayjkaiaawMcaaaaacqGHsislcqaIXaqmaiaawIcacaGLPaaacqGGSaalaaa@4823@

where μ_0 _and μ_1 _are allocation parameters, and *I*_*m*_*(t,h) *is the simulated value of the absorbed relative photosynthetically active radiation (PAR) intensity averaged over the entire leaf area of a single plant of height *h *at time *t*. From eqn. (7), it is apparent that the allocation relationship is dependent on the light environment within a forest stand. Hence, a larger μ_1 _might enhance the height growth of smaller plants in a dark light environment inside the forest, consequently reducing differences in growth rate between trees of different height classes. Thus, these parameters can be used as a measure of competition for light between trees [49]. The general outline and model components of MINoSGI are described in detail by Watanabe *et al*. [28].

### 2. Soil carbon dynamics

The net carbon uptake in a forest ecosystem, net ecosystem production (NEP), is given as

*NEP *= (*GPP *- *R*_*a*_) - *R*_*h *_= *NPP *- *R*_*h*_,

where *R*_a _and *R*_h _are autotrophic and heterotrophic respiration rates, respectively. *R*_a _is a carbon loss by a plant, the sum of growth respiration and maintenance respiration of nonphotosynthetic (sapwood and fine roots) organs [33]. In contrast, *R*_h _is the loss of carbon accompanying with the activity of soil microorganisms that decompose soil organic matter (SOM). In the original MINoSGI, *R*_h _was given as a function of soil temperature without considering carbon cycling in the soil [28]. Here, we incorporated a below-ground soil carbon dynamics model into MINoSGI and *R*_h _is now modelled through the decomposition processes of soil organic carbon related with the above-ground leaf fall event. The framework of the soil carbon dynamics is based on [2]. Litter and two SOM pool compartments, each of which has a different decomposition rate, were considered. Decomposition of each pool is calculated as a function of soil temperature and moisture at hourly time steps. A fraction of decomposed litter is released to the atmosphere and the remaining enters the SOM pools. The allocation rate to each SOM pool is given as a constant. Then, *R*_h _is obtained as the summation of respiration rates from each compartment. All parameters used in the soil carbon dynamics model are the same as in [2].

### 3. Description of the deciduous canopy development model

**Figure 6:**
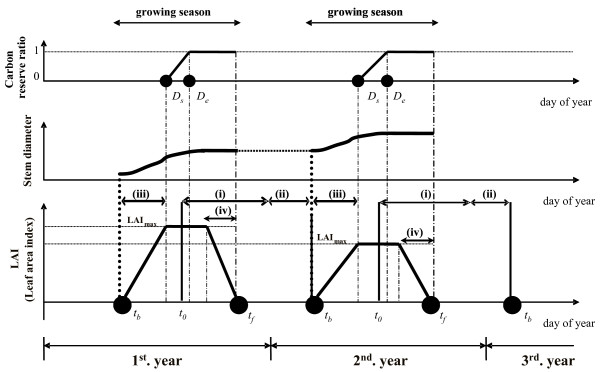
Schematic diagram of the seasonal courses of LAI, radial growth and the carbon reserve ratio with phenological events for a deciduous tree. *t*_*0*_, *t*_*b *_and *t*_*f *_are the onset of bud dormancy, the timing of bud burst and the completion of leaf fall, respectively. (i) is the combined duration of autumn and winter dormancy. The duration of (ii) is the ontogenesis development stage toward bud burst. In addition, the duration of (iii) and (iv) are the stages of LAI increment from bud burst to its maximum and LAI decrement from the maximum to the completion of leaf fall. The timings *D*_*s *_and *D*_*e *_mean the onset and end, respectively, of carbon reservation in the storage pool of woody nonphotosynthetic organs. For simplicity, *D*_*s *_was assumed to be identical to *D*_*e *_(i.e., *D*_*s *_= *D*_*e *_= *D*_*x*_) in this study.

The scheme of processes incorporated into the present deciduous canopy development model is shown in Fig. 6. The canopy development model is composed of a phenological model and a model for maximum leaf area index based on the carbon reserves in woody tissues.

The phenological model describes the process of chilling requirement during dormancy that must be fulfilled before the ontogenetic development toward bud burst commences, and determines the timing of bud burst through the stage of ontogenetic development after the release of dormancy [50]. In Fig. 6, *t*_*b *_and *t*_*f *_indicate the dates of bud burst and completion of leaf-fall, respectively. Duration (i) is the development stages of bud dormancy period (autumn-winter dormancy) starting at *t*_0 _(thus buds growing in the next year are assumed to be formed before *t*_0_), where we define 'dormancy' as a state in which buds do not grow even under favorable ambient conditions. Normally, the dormancy processes between autumn and winter are different. Thus, each has a different response function of temperature [15,51,52]. However, in recent studies, the same developmental response function of temperature has been used consistently through the autumn to winter periods [15]. (ii) is a stage during the ontogenetic development of buds toward bud burst after duration (i) has been completed. (iii) and (iv) are the increment and decrement processes of the leaf amount in a year, respectively. *D*_*s *_represents the date on which trees start to store part of surplus assimilates in the tree body, and *D*_*e *_the date after which all the surplus assimilates are stored without being used for tree growth. In the present study, we assumed to be *D*_*s *_= *D*_*e*_≡*D*_*x *_and treated *D*_*x *_as a fixed date. The outline of the above-mentioned processes is specified in the following. Details on the treatment of *D*_*s *_and *D*_*e *_are described later.

To determine the timing of bud burst, approaches proposed by [51,52] were used: these include the determination of the stage of dormancy to describe the chilling-requiring stage indispensable for the release from autumn-winter dormancy, and the stage of ontogenesis essential for the induction of bud burst after the autumn-winter dormancy period.

The time change of the stage of dormancy *S*_*D*_*(t) *can be written in the form aggregated with time *t *using the rate of bud development *f*_*D*_*(t) *as follows:

SD(t)=∫t0tfD(t)dt,
 MathType@MTEF@5@5@+=feaafiart1ev1aaatCvAUfKttLearuWrP9MDH5MBPbIqV92AaeXatLxBI9gBaebbnrfifHhDYfgasaacH8akY=wiFfYdH8Gipec8Eeeu0xXdbba9frFj0=OqFfea0dXdd9vqai=hGuQ8kuc9pgc9s8qqaq=dirpe0xb9q8qiLsFr0=vr0=vr0dc8meaabaqaciaacaGaaeqabaqabeGadaaakeaacqWGtbWudaWgaaWcbaGaemiraqeabeaakmaabmaabaGaemiDaqhacaGLOaGaayzkaaGaeyypa0Zaa8qmaeaacqWGMbGzdaWgaaWcbaGaemiraqeabeaakmaabmaabaGaemiDaqhacaGLOaGaayzkaaGaemizaqMaemiDaqhaleaacqWG0baDdaWgaaadbaGaeGimaadabeaaaSqaaiabdsha0bqdcqGHRiI8aOGaeiilaWcaaa@4295@

where *t*_0 _is the beginning of the autumn-winter dormancy. Häkkinen, Linkosalo and Hari [14] applied the dependence of *f*_*D*_*(t) *on temperature *T(t) *based on experiments by [52] to seedlings and seeds of birch *(Betula pubescens)*, and *f*_*D*_*(t) *= *f*_*D*_*(T(t)) *was described as

fD(T(t))={0T(t)≤Tm or T(t)≥TMT(t)−TmTo−TmT(t)>TmT(t)−TMTo−TMT(t)<TM
 MathType@MTEF@5@5@+=feaafiart1ev1aaatCvAUfKttLearuWrP9MDH5MBPbIqV92AaeXatLxBI9gBaebbnrfifHhDYfgasaacH8akY=wiFfYdH8Gipec8Eeeu0xXdbba9frFj0=OqFfea0dXdd9vqai=hGuQ8kuc9pgc9s8qqaq=dirpe0xb9q8qiLsFr0=vr0=vr0dc8meaabaqaciaacaGaaeqabaqabeGadaaakeaacqWGMbGzdaWgaaWcbaGaemiraqeabeaakmaabmaabaGaemivaq1aaeWaaeaacqWG0baDaiaawIcacaGLPaaaaiaawIcacaGLPaaacqGH9aqpdaGabeqaauaabaqadiaaaeaacqaIWaamaeaacqWGubavdaqadaqaaiabdsha0bGaayjkaiaawMcaaiabgsMiJkabdsfaunaaBaaaleaacqWGTbqBaeqaaOGaeeiiaaIaem4Ba8MaemOCaiNaeeiiaaIaemivaq1aaeWaaeaacqWG0baDaiaawIcacaGLPaaacqGHLjYScqWGubavdaWgaaWcbaGaemyta0eabeaaaOqaamaalaaabaGaemivaq1aaeWaaeaacqWG0baDaiaawIcacaGLPaaacqGHsislcqWGubavdaWgaaWcbaGaemyBa0gabeaaaOqaaiabdsfaunaaBaaaleaacqWGVbWBaeqaaOGaeyOeI0Iaemivaq1aaSbaaSqaaiabd2gaTbqabaaaaaGcbaGaemivaq1aaeWaaeaacqWG0baDaiaawIcacaGLPaaacqGH+aGpcqWGubavdaWgaaWcbaGaemyBa0gabeaaaOqaamaalaaabaGaemivaq1aaeWaaeaacqWG0baDaiaawIcacaGLPaaacqGHsislcqWGubavdaWgaaWcbaGaemyta0eabeaaaOqaaiabdsfaunaaBaaaleaacqWGVbWBaeqaaOGaeyOeI0Iaemivaq1aaSbaaSqaaiabd2eanbqabaaaaaGcbaGaemivaq1aaeWaaeaacqWG0baDaiaawIcacaGLPaaacqGH8aapcqWGubavdaWgaaWcbaGaemyta0eabeaaaaaakiaawUhaaaaa@79C8@

with the optimal chilling temperature, *T*_*o *_(*T*_*o *_= 6.9), while *T*_*m *_and *T*_*M *_are constants (*T*_*m *_= -3.4, *T*_*M *_= 10.4) [17]. On the other hand, the stage of ontogenesis *S*_*o*_*(t) *is described by the rate of ontogenetic development, *f*_*o*_*(t) *based on [51] ([14]) as follows:

So(t)=∫tftfo(t)dt,
 MathType@MTEF@5@5@+=feaafiart1ev1aaatCvAUfKttLearuWrP9MDH5MBPbIqV92AaeXatLxBI9gBaebbnrfifHhDYfgasaacH8akY=wiFfYdH8Gipec8Eeeu0xXdbba9frFj0=OqFfea0dXdd9vqai=hGuQ8kuc9pgc9s8qqaq=dirpe0xb9q8qiLsFr0=vr0=vr0dc8meaabaqaciaacaGaaeqabaqabeGadaaakeaacqWGtbWudaWgaaWcbaGaem4Ba8gabeaakmaabmaabaGaemiDaqhacaGLOaGaayzkaaGaeyypa0Zaa8qmaeaacqWGMbGzdaWgaaWcbaGaem4Ba8gabeaakmaabmaabaGaemiDaqhacaGLOaGaayzkaaGaemizaqMaemiDaqhaleaacqWG0baDdaWgaaadbaGaemOzaygabeaaaSqaaiabdsha0bqdcqGHRiI8aOGaeiilaWcaaa@43A8@

where *t*_*f *_is the beginning of the stage of ontogenesis. In addition, *f*_*o*_*(t) *is given as

*f*_*o*_(*t*) = *g*_*o*_(*T*(*t*))·*w*_*o*_(*L*(*t*)),

where *g*_*o*_*(T(t)) *denotes the dependence of the rate of ontogenesis on temperature as follows [51,17]

go(T(t)){=0T(t)>0=28.41+e−0.185(T(t)−18.4)T(t)>0,
 MathType@MTEF@5@5@+=feaafiart1ev1aaatCvAUfKttLearuWrP9MDH5MBPbIqV92AaeXatLxBI9gBaebbnrfifHhDYfgasaacH8akY=wiFfYdH8Gipec8Eeeu0xXdbba9frFj0=OqFfea0dXdd9vqai=hGuQ8kuc9pgc9s8qqaq=dirpe0xb9q8qiLsFr0=vr0=vr0dc8meaabaqaciaacaGaaeqabaqabeGadaaakeaacqWGNbWzdaWgaaWcbaGaem4Ba8gabeaakmaabmaabaGaemivaq1aaeWaaeaacqWG0baDaiaawIcacaGLPaaaaiaawIcacaGLPaaadaGabeqaauaabeqacmaaaeaacqGH9aqpaeaacqaIWaamaeaacqWGubavdaqadaqaaiabdsha0bGaayjkaiaawMcaaiabg6da+iabicdaWaqaaiabg2da9aqaamaalaaabaGaeGOmaiJaeGioaGJaeiOla4IaeGinaqdabaGaeGymaeJaey4kaSIaemyzau2aaWbaaSqabeaacqGHsislcqaIWaamcqGGUaGlcqaIXaqmcqaI4aaocqaI1aqndaqadaqaaiabdsfaunaabmaabaGaemiDaqhacaGLOaGaayzkaaGaeyOeI0IaeGymaeJaeGioaGJaeiOla4IaeGinaqdacaGLOaGaayzkaaaaaaaaaOqaaiabdsfaunaabmaabaGaemiDaqhacaGLOaGaayzkaaGaeyOpa4JaeGimaadaaaGaay5EaaGaeiilaWcaaa@5DE2@

and *w*_*o*_*(L(t)) *means the effect of the light conditions (such as light intensity, night length, and spectral composition of light) on ontogenesis. *L(t) *denotes the signal from light conditions [14]. Changes in light conditions during the annual cycle are the most reliable source of information reflecting the timing of seasons, especially in the boreal zone because these seasonal changes are much larger than those in lower latitude areas and the photoperiod plays a role in controlling the timing of phenological events of various boreal tree species [14,15]. The effect of these changes was considered in the present case of cool-temperate climate area, i.e. we assumed that *w*_*o*_*(L(t)) *is given by

wo(L(t)){=0L(t)<Swt=1L(t)>Swt,
 MathType@MTEF@5@5@+=feaafiart1ev1aaatCvAUfKttLearuWrP9MDH5MBPbIqV92AaeXatLxBI9gBaebbnrfifHhDYfgasaacH8akY=wiFfYdH8Gipec8Eeeu0xXdbba9frFj0=OqFfea0dXdd9vqai=hGuQ8kuc9pgc9s8qqaq=dirpe0xb9q8qiLsFr0=vr0=vr0dc8meaabaqaciaacaGaaeqabaqabeGadaaakeaacqWG3bWDdaWgaaWcbaGaem4Ba8gabeaakmaabmaabaGaemitaW0aaeWaaeaacqWG0baDaiaawIcacaGLPaaaaiaawIcacaGLPaaadaGabeqaauaabeqaciaaaeaacqGH9aqpcqaIWaamaeaacqWGmbatdaqadaqaaiabdsha0bGaayjkaiaawMcaaiabgYda8iabdofatjabdEha3naaBaaaleaacqWG0baDaeqaaaGcbaGaeyypa0JaeGymaedabaGaemitaW0aaeWaaeaacqWG0baDaiaawIcacaGLPaaacqGH+aGpcqWGtbWucqWG3bWDdaWgaaWcbaGaemiDaqhabeaaaaaakiaawUhaaiabcYcaSaaa@4E35@

where *Sw*_*t *_is daily mean solar radiation. Häkkinen, Linkosalo and Hari [14] used calendar date as an operational variable for light conditions. Instead of calendar date, we used *Sw*_*t *_and assumed to be *w*_*o*_*(L(t)) *= 1 when *Sw*_*t *_was greater than 300 Wm^-2 ^which can be obtained for the period from the middle of February to the end of October in the present study site under sunny weather conditions.

Among the above functions, the unknown variables necessary to be established are the dates of the beginning and end of the autumn-winter dormancy, and a decision needs to be made as to when ontogenetic development of buds toward bud burst is completed. Normally, the onset of autumn-winter dormancy is likely to vary with species and annual environmental variables, and should therefore be estimated using an experimental approach [51] or by statistical interpretation using long-term phenological data [14]. In this article, it is assumed that the beginning of the autumn-winter dormancy in the process (i) in Fig. 6 occurs on the same fixed date each year as in [52]. While the end of the autumn-winter dormancy is described using the accumulated sum of the value of *S*_*D*_(t) showing the state of dormancy obtained from the response function of air temperature, the stage is completed when its accumulated sum surpasses an assigned threshold value (*D*_*crit*_). The process in (ii) is also determined by the sum of values showing the developmental process of the bud toward bud burst, calculated by the logistic response function of air temperature just after the dormancy period (i). Bud burst occurs when *S*_0_(t) in Eqns. (11) exceeds a given threshold value (*B*_*crit*_). These threshold values assigned in Eqns. (9) and (11) to determine the dates of the end of the autumn-winter dormancy and bud burst are established based on the temperature-dependence of leaf opening obtained from inventory data in Japanese larch site (*L. kaempferi *spp.) by [53] (see Table 1).

**Table 1:** The parameter list for the *Larix kaempferi *stand.

Symbol	Definition	Value	Unit	Reference or adjusted in the present study
V_m_	Maximum catalytic activity of Rubisco	35	μmol m^-2 ^s^-1^	Kurachi (1986)
α	Allometric parameter	0.022	kg cm^-2 ^m^-1^	Kurachi (1986)
θ	Allometric parameter	0.0256	m^2 ^cm^-2^	Kurachi (1986)
SLA	Specific leaf area	16.7	m^2 ^kg^-1^	Kurachi (1986)
η	Allometric parameter	1.9	-	adjusted in eqn.(3), (4)
δ	Factor of crown diameter	4.0	m cm^-1^	adjusted in eqn.(5)
f_d_	Ratio of leaf respiration to Vm	0.017	-	adjusted in eqn.(6)
μ_0_	Parameter for light competition	0.3	-	adjusted in eqn.(7)
μ_1_	Parameter for light competition	0.5	-	adjusted in eqn.(7)
D_x_	Parameter for the start of carbon reserves	230	-	
*D*_*crit*_		50		This issue (fixed)
*B*_*crit*_	Parameter for threshold value to determine the date of bud burst	80		This issue (fixed)

In addition, the incremental process (iii) of leaf amount after bud burst until LAI is at a maximum change with the thermal condition in the current year. We use the growing degree-day as an index of the thermal condition which is defined as the summation of the canopy-effective temperature (*T*_*ce*_), where *T*_*ce *_is given by the subtraction of the base temperature (herein, the base temperature is set to 5°C) from the canopy-averaged temperature. The canopy-averaged temperature is estimated by dividing the summation of a product of leaf amount and temperature at each vertical layer obtained from multilayered meteorological model by total leaf amount over a canopy. When the accumulated temperature is greater than an assigned threshold value which is set to 250°C [54,55], LAI reaches a maximum. On the other hand, leaf-fall in autumn through duration (iv) is described using a 10-day running mean temperature calculated from the daily mean temperature, and the start of leaf-fall is determined when the 10-day value is less than an assumed threshold value, and all leaves fall within 30 days.

### 4. Contribution of carbon reserve to the maximum LAI- a maximum LAI model

The leaf amount of a tree has a close allometric relationship with the stem diameter at the bottom of the crown height (the pipe model; [47]) as denoted in eqn.(4). However, it is likely that the leaf amount of a deciduous tree is not only related to the stem diameter but also closely to the carbohydrate reserve in the trees (e.g. [56,57]). Determination of the maximum leaf amount (i.e., maximum LAI) is greatly affected by both the environmental variables of the current year and the carbon storage of the previous year which in turn reflects the environmental conditions of the previous year. Therefore, the decisions of *D*_*s *_and *D*_*e *_in Fig. 6 (when part of surplus production starts being stored in the tree body, and when all surplus production starts being stored) allow us to make explicit the effect of environmental differences on the annual amount of carbon storage.

The instant that bud burst occurs, deciduous trees start photosynthesizing with their unfolded leaves in the current year. The increase of leaf amount in spring, however, is likely to be so rapid that photosynthesis by leaves is not enough to support the rapid growth rate [45]. Hozumi and Kurachi [57] investigated the relationship between the relative growth rate of opened leaves and the seasonality of leaf biomass production using a component model approach, and suggested that the total growth rate of leaf biomass in spring was largely supported by the carbon supply from non-photosynthetic organs where carbon in the previous year is stored. Moreover, Hozumi and Kurachi [57] found that leaf amount was increased due to both the contributions of carbohydrates reserved in the previous year and net assimilates of unfolded leaves in the current year through the seasonal period until the leaf amount is at maximum.

To combine the effect of carbon stores and the pipe model theory as concomitant factors determining the leaf amount of a tree, the following was assumed. If the carbohydrates are abundant (i.e., if the dry weight of carbohydrates reserved in the tree body is greater than the leaf dry weight of the tree predicted from the stem diameter through the allometric relationship of the pipe model), the maximum possible leaf amount of a tree is equivalent to the predicted value. But if the carbohydrates are not enough, realized leaf amount of a tree is smaller than the one predicted by the pipe model. It should be noted here that the dates of *D*_*s *_and *D*_*e *_in Fig. 6 is important to explicitly include the effects of environmental variations on the amount of carbon storage, because these dates determine the length of duration during which surplus production is accumulated as carbon stores. Hence realized LAI of the forest may also be affected by the carbon storage of the trees in the previous year, which in turn shows that the environmental conditions of the previous year affects the stand LAI in the current year. Actually, LAI of a forest can vary from year to year with climatic conditions and environmental events.

As mentioned above, it is incidentally known that part of the carbon resources is also used for spring woody production [45] and reproductive organs. Moreover, the stored carbohydrate (starch) might be used for active radial (or cambial) division (new cell-wall formation) before the full development of leaves after bud burst [58]. However, we ignored such processes in this study to simplify the relationship between the leaf amount and the carbon resources. The assumption that leaf growth comes only from the carbohydrate reserve while plant growth comes only from newly assimilated carbon might be difficult to apply to all tree species. For instance, it might be true of diffuse porous trees like birch, whilst not necessarily true of ring porous trees like oak where spring wood growth must start before bud burst to support leaf growth [45]. The observational result on the translocation from carbon reserves in spring of *L. kaempferi *species by [57] might support the treatment on the carbon translocation as part of ring porous trees, although *L. kaempferi *does not belong to them. Nevertheless, in this study, we employed this assumption to describe explicitly the possible relationship between maximum LAI and carbon reserves.

Aber and Federer [19] developed a forest carbon and water balance model (PnET) to describe monthly time-steps of water and carbon balances in a forest, and Aber *et al*. [18] further improved the original model (PnET-II), in which bud and wood carbon storage pools accumulated on a monthly basis during several years before the current year were used to simulate the foliar and wood production for the next year, respectively. Maximum leaf amount (i.e. LAI) for the next year was then decided, and PnET-II with the routine of carbon storage was applied to hardwood, spruce-fir, mixed hardwood/spruce-fir and mixed hardwood/pine forest sites [18]. In the present study, we used *L. kaempferi *which has short and long branches. In general, leaf primordium of *L. kaempferi *species during the middle of growing season in a given year is likely to perform on a short branch for leaf production in the following year, and the formation of leaf primordium of *Larix *tree species such as *L. kaempferi *and *Larix gmelini *appears to be largely affected by varying meteorological conditions before the onset of the formation (personal communication with Dr. Uchiyama from her unpublished data). After the leaf expansion in the next year, most of the carbon assimilation by opened leaves on short branches is provided for the growth of long branches in the corresponding year. Additionally, leaf primordium is formed on long branches, and new leaves from the long branches occur after all leaves from leaf primordium on short branches are opened. It implies that most of carbon assimilation by the leaves in the current year might be used to construct additional buds and wood production in the year along with the rest of carbon reserves stored in the previous year. However, it is difficult to separate how carbon amount from reserves in a previous year and gained by opened leaves in the current year to produce wood and foliar growth has been used. In our study, it was assumed that the production of leaves in the current year depended on the carbohydrates from the previous year which contribute to the entire leaf amount (i.e. maximum LAI) over a stand, including leaf amount from the secondary wood production (long branches) produced by the opened leaves on short branches. Further, we assumed that carbon assimilation by leaves produced in spring is used for plant buds and wood growth in the current year after bud burst. With these simple treatments and assumptions to link the woody carbon storage with the maximum LAI, we modeled phenological processes of deciduous *L. kaempferi*.

### 5. Modeling carbon storage in association with seasonal carbohydrate mobilization

After the leaf amount reached a maximum, part of the assimilated carbon may continue to be utilized in further growth. The rest of the carbon may possibly be transformed into carbohydrates such as sugar (including glucose, fructose and sucrose), and starts to be stored as the carbon pool in the woody tissues and bark of branches, trunks and roots during the periods of low photosynthesis to fuel the maintenance of respiration, especially during autumn [59]. These surplus photosynthetic products are stored until the end of the growing season [57]. Seasonal reserve mobilization of carbohydrates has been described in several types of trees and for different stand ages [45,56] and the seasonality has been quantitatively investigated (e.g., *Betula ermanii *by [60]). Hence we assumed that reserved carbohydrates are used for respiration of trees in leafless seasons and construction of leaves in the next spring.

The total amount of carbon stored in the tree body is affected by the timing of growth cessation of trees, as stated before. Stem-diameter growth (radial growth) often continues for a long time after shoot elongation ceases in late summer to autumn [61]. In addition, the seasonal pattern of shoot elongation and diameter growth in *B*. *ermanii *was observed during the growing season over two years (2003–2004) in Hokkaido, north of Japan, and it was found that cessation of shoot elongation was earlier than that of radial growth (unpublished data by M. Toda, K. Kato and K. Laska). Moreover, the timing of diameter growth cessation was dependent on tree size (tree height), but it mostly occurred around the beginning of September in both years. The tendency was consistent with the report by [53], which showed that diameter growth in *L. kaempferi *stopped around the end of August or the beginning of September. Thus, in the present model, it was assumed that all surplus assimilated carbon after cessation of diameter growth is stored in the body of the tree. As for the dates of *D*_*s *_and *D*_*e*_, however, we assumed in the present paper that all the surplus production starts being stored on a certain day of year (i.e., *D*_*s *_= *D*_*e*_≡*D*_*x *_in Fig. 6), and treated *D*_*x *_as a fixed date. For estimating *D*_*x*_, we simply referred to the end of diameter growth based on the observational fact that the diameter growth of *L. kaempferi *stops around the end of August or the beginning of September [53]. We treated *D*_*x *_as an adjusted parameter in this study and determined it as follows. For a given range of *D*_*x*_, the size distribution of plant height class was simulated together with the adjusted parameters in eqn.(5), (6), (7); *D*_*x *_was then optimized by finding *D*_*x *_that gives the best agreement between the observed and simulated size distributions.

### 6. Ecological inventory and meteorological data sets

Ecological inventory data from 1982 to 1989 used for the model validation were obtained from a plantation of Japanese larch (*Larix kaempferi *(Lamb.) Carrière), a deciduous tree species, in the Nagoya University Experimental Forest at Inabu, Aichi Prefecture in central Japan. Total precipitation during the growing season from April to October was 1494 mm and the mean air temperature was 7.6°C over the three years from 1982 to 1984 [62]. The study plot of 200 m^2 ^(20 m × 10 m, 35°11'N, 137°33'E, 1040 m a.s.l.) was established in 1982 [63]. In 1982, stand age was 18 years. Tree density was 2580, 2529 and 2327 trees ha^-1 ^in 1982, 1983 and 1984, respectively. The mean height of the trees, the mean stem diameter at breast height, and the mean stem diameter at the lowest branch height increased from 10.3 m to 11.7 m, 10.7 cm to 11.8 cm, 5.2 cm to 6.0 cm during the three years, respectively. More details are described in [62].

Meteorological data are required as a set of forcing data for MINoSGI. Unfortunately, simultaneous observation procedures were not established with the above-mentioned ecological inventory data. Hence, use was made of a specified data source from the AMeDAS (Automated Meteorological Data Acquisition System) at the station at Inabu (35°12.6'N, 137°30.6'E, 505 m a.s.l.) 5.7 km away from the site. The same data correction for altitude as used by [28] was applied to obtain air temperature and pressure with altitude assuming a moist adiabatic lapse rate of 0.005 Km^-1^, and humidity calculated from the corrected variables. Because the present study site is very close to the site used by [28], the other meteorological variables used in the present study were the same as those described by [28].

### 7. Simulation setup

The inventory data include the number of trees, the tree height (*h*) and stem diameter at breast height (*D*_*BH*_) and at the lowest branch height (*D*_*B*_) and the total weight (*w*) for an individual tree. Using time steps of one year for the size dynamics model and one hour for the microclimate model, simulation over seven years from 1982 to 1989 was carried out. Thus, structural changes of the forest were derived at yearly intervals. However, leaf photosynthesis, calculated on the basis of a combination of the photosynthesis at the biochemical level [64], the stomatal conductance of the semi-empirical model proposed by [65], the respiration at a leaf scale in eqn. (6) together with the woody and root respiration in the microclimate model, can be calculated hourly for an area-averaged and multi-layered space. Therefore, the framework enables us to estimate the seasonal production at daily intervals, which leads to the determination of phenological events in the spring of the subsequent year as specified in Methods. The inventory data from 1982 were used as the initial input data, and seven-year simulated outputs were compared with the observational data for each year. The simulation model was given boundary conditions of tree height class with zero flux at the lowest boundary and no outflow flux at the top boundary, during the simulation period. These living trees were grouped at 1-m intervals into height classes from 0 m to 30 m for each year. In order to produce a proper calculation, the closure of annual carbon balance has been examined for each one-year time interval.

The species-specific model parameters in MINoSGI obtained from the inventory data in the study plot are listed in Table 1. In addition, the adjusted parameters are also summarized. In the MINoSGI simulation, we used the same model parameters for respiration of stem and fine roots of *L. kaempferi *as those of *C. japonica *used in the previous research [28] because of the lack of inventory data for these parameters. Hukami [66] and Hagihara and Hozumi [67] investigated the difference in the annual respiration rates of stem and fine roots between *L. kaempferi *and *C. obtusa *(which is one of typical coniferous species and has eco-physiological characteristics similar to *C. japonica*) at the stand level. Hukami showed that stem and fine root respiration rates of 16- to 24-year-old *L. kaempferi *were 4.2–8.4 and 4.3–5.4 ton ha^-1 ^year^-1^, respectively. Hagihara and Hozumi [67] showed that those of 18-year-old *C. obtusa *were 9.9 and 4.6 ton ha^-1 ^year^-1^. Therefore, we assumed that the difference in stem respiration rate between these species does not largely affect the production variables over seven years during the present simulation period because stand age of *L. kaempferi *during the seven years is 18 to 25 years. All of the model parameters of except for those given in Table 1 were therefore the same as in [28]. Furthermore, we determined initial carbon stocks in litter and two SOM pools in soil carbon dynamics model based on the soil respiration measurement in *C. japonica *stand in the Inabu experimental field [67]. Initial litter stock was 3.1 tC ha^-1^. On the other hand, values of the reminder initial SOM C stocks were given as 6.0 tC ha^-1 ^in intermediate and 7.6 tC ha^-1 ^in slow SOM pools, respectively, from estimation of mineral soil and root components by [67].

### 8. Determination of model parameters and validation

Some unknown parameter values were adjusted by simulation because these were difficult to obtain from observational approaches or due to a lack of observations. Of the unknown parameters described in Table 1, the four ones treated in [28] were investigated; δ in eqn. (5), *f*_*d *_in eqn. (6), and *μ*_0 _and *μ*_1 _in eqn. (7) (see Methods section). The value of η in eqns. (3) and (4) was also treated as one of the adjusted parameters. In addition, as described in Methods section, the additional parameter that characterizes deciduous trees in conjunction with phenological events was simultaneously adjusted with the above-mentioned variables.

**Figure 7:**
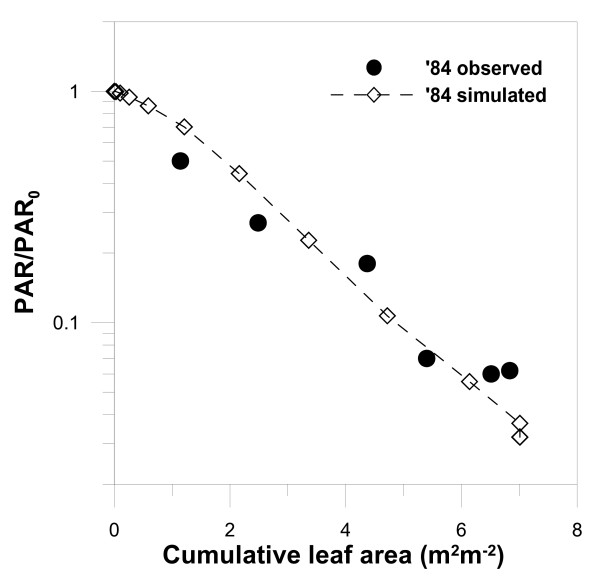
Observed and simulated vertical profiles of relative intensity of photosynthetically active radiation (PAR/PAR_0_) against cumulative leaf area per unit ground area in the *Larix kaempferi *stand in 1984. PAR in each layer is normalized by PAR at the canopy top (PAR_0_). Six observed data were obtained within the stand in each year.

**Figure 8:**
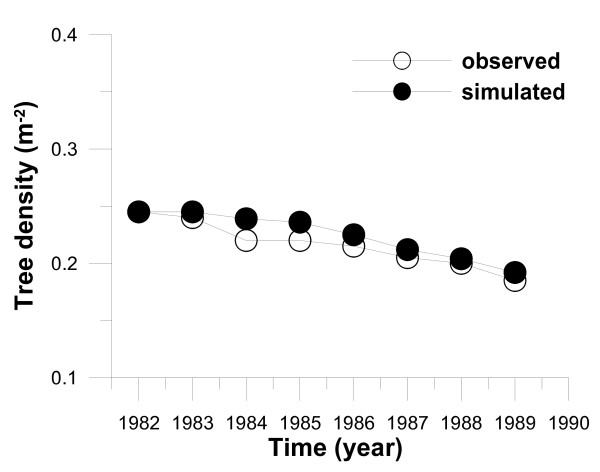
Observed and simulated annual changes in tree density of the *Larix kaempferi *stand from 1982 to 1989.

**Figure 9:**
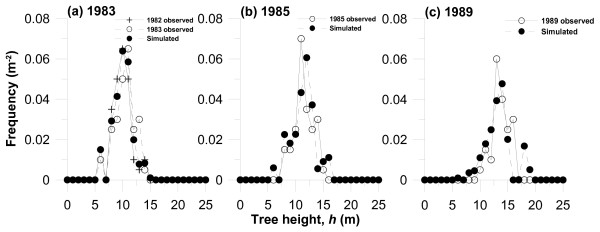
Observed and simulated frequency distributions of *Larix kaempferi *trees in each height class in (a) 1983, (b) 1985 and (c) 1989. All live trees were classified in tree height at 1-m intervals. The vertical axis denotes the number of trees in each tree height per study area (200 m^2^). In Figure 9 (a), the initial size distribution in 1982 is also presented.

For instance, the parameter *f*_*d *_affects the time change of stand-level variables such as tree density, averaged tree height, LAI, net primary production (NPP), biomass and size distribution of tree height. Larger *f*_*d *_brings about an increase in leaf respiration and therefore may increase the mortality of smaller-sized trees, which generally gain lower carbon assimilation due to the low light intensity they receive in the forest. On the other hand, the time change of size distribution and LAI did not always depend on *f*_*d *_alone. The simulated size distribution is largely dependent on the height-diameter growth relationship parameters (*μ*_0 _and *μ*_1_), which change the light environment within the forest. In addition, the timings of the start of carbon reservation (*D*_*x*_) may have an effect on LAI and size distribution. Thus, an interactive adjustment between these parameters should be made for their final estimation by comparing the simulated and observed results of vertical profile of light intensity within a canopy (Fig. 7), tree density (Fig. 8), LAI (Fig. 2(a)) and size distribution (Fig. 9).

Initially, we assumed that the crown of *L. kaempferi *is parabolic-shaped. The parameters δ, η were determined when the simulated vertical profile of light intensity with depth into the canopy is consistent with the hourly-averaged measurement on photosynthetically active radiation (PAR) observed on specific days in 1984 by [34]. In this operation, we gave a constant allocation parameter set of *μ*_0 _= 0.5, *μ*_1 _= 0.5, *f*_*d *_= 0.017 and *D*_*x *_= 200. *μ*_0 _and *μ*_1 _may be involved in the change in light intensity within a canopy, but their effect on light intensity was very small compared to those of δ and η. When the combination of δ = 4.0 m cm^-1 ^and η = 1.9 was chosen, the simulated and observed values were highly correlated with R^2 ^= 0.83. The magnitude of parameter δ represents the ratio of the ground-level crown diameter to the ground-level stem diameter for an isolated plant [28], and we obtained a ratio of 40. The value of η = 1.9 represents the canopy shape with widely vertical-attached leaves from the bottom to the top of an isolated tree [48], and the crown shape agreed with the vertical shape of a *L. kaempferi *tree.

Furthermore, *f*_*d *_was adjusted so that the simulations would reproduce the annual live and dead tree numbers well (Fig. 8), both of which are the consequence of competition between trees with the progress of time. The value of *f*_*d *_was estimated by parameter adjustment as *f*_*d *_= 0.017, which is similar to that of *C. japonica *(*f*_*d *_= 0.022) [28]. In this operation, we also gave a constant allocation parameter set of *μ*_0 _= 0.5, *μ*_1 _= 0.5 and *D*_*x *_= 200. Simultaneously, with the parameter set, good agreement between the observed and simulated maximum LAI in 1982 and 1984 was obtained (Fig. 2(a)) (at a particular time during the late growth period each year; LAI observations of individual trees were carried out by [34]). Kurachi [34] estimated the value of LAI by using observed values of leaf biomass and specific leaf area (SLA) in each vertical layer at 1-m intervals. Unfortunately, Kurachi [34] did not measure SLA in upper layers near the top of the canopy in 1983, and used SLA values measured in the lower layers to complement SLA in the upper layers. However, we found that the complemented SLA values in the upper layers in 1983 were smaller than the observed upper SLA values in 1982 and 1984. Therefore, we believe that LAI in 1983 obtained by [34] was underestimated because the underestimated SLA values were used (Fig. 2(a)). We assumed that the additional parameter *D*_*x *_should be indispensable to get a final determination of *f*_*d*_. However, we found that the impact of the difference in *f*_*d *_was larger compared with that in *D*_*x*_. Then, *D*_*x *_was adjusted to DOY 200 (day of year) for every year, and the observed and simulated dates of bud burst over 3 years from 1982 to 1984 were 12 April and 12 April, 26 April and 6 May, 15 April and 14 April, respectively.

Finally, we adjusted tuning parameters *μ*_0 _and *μ*_1 _so that the simulated size distribution of tree height would be consistent with the observed results. As shown in eqn. (7), greater *μ*_0 _brings about a greater increase in tree growth in the vertical direction compared with that towards the lateral diameter direction, whilst a greater value of *μ*_1 _a greater height increment in the smaller-sized tree growth in lower light intensity within the forest. The values of *μ*_0 _and *μ*_1 _obtained in this study were 0.3 m cm^-1 ^and 0.5 m cm^-1^. *μ*_1 _was extremely large in comparison with that of *C. japonica *obtained by [28]. Although the estimation of the parameters was simply made so as to achieve agreement between the observed and simulated results, differences in the same parameter between tree species may be related to the plant functional type, e.g., shade-intolerant species such as deciduous *L. kaempferi *may have enhanced height growth when the tree is small under a canopy, whereas allocation to height growth in *C. japonica*, which is shade-tolerant, may be less pronounced. The difference in the adjusted parameter *f*_*d *_may also affect the size distribution. However, we found that the difference in *f*_*d *_does not greatly change the size distribution.

## Competing interests

The author(s) declare that they have no competing interests.

## Authors' contributions

MT carried out numerical experiments and drafted the manuscript. MT, MY and TH conceived of this study, participated in its design and coordination; AS and TW helped to draft the manuscript. All authors read and approved the final manuscript.
